# The complete chloroplast genome sequence of *Santalum album*

**DOI:** 10.1080/23802359.2019.1704199

**Published:** 2020-01-07

**Authors:** Dejun Yang, Qiong Qiu, Linhong Xu, Yumei Xu, Yi Wang

**Affiliations:** a Institute of Tropical Forestry, Yunnan Academy of Forestry, Puwen, People's Republic of China;; b Laboratory of Forest Plant Cultivation and Utilization, Yunnan Academy of Forestry, Kunming, People's Republic of China

**Keywords:** *Santalum album*, chloroplast, Illumina sequencing, phylogenetic analysis

## Abstract

The first complete chloroplast genome (cpDNA) sequence of *Santalum album* was determined from Illumina HiSeq pair-end sequencing data in this study. The cpDNA is 144,101 bp in length, contains a large single copy region (LSC) of 83,796 bp and a small single copy region (SSC) of 11,277 bp, which were separated by a pair of inverted repeats (IR) regions of 24,514 bp. The genome contains 123 genes, including 80 protein-coding genes, 8 ribosomal RNA genes, and 35 transfer RNA genes. The overall GC content of the whole genome is 38.0%, and the corresponding values of the LSC, SSC, and IR regions are 35.9%, 31.4%, and 43.1%, respectively. Further phylogenomic analysis showed that *S. album* and *Osyris alba* clustered in a clade in Santalales order.

*Santalum album* is the species within the family Santalaceae. It is widely distributed in India, Malaysia, and Australia, and commonly known as sandalwood (Kim et al. 2005). The essential oil of sandalwood widely used in perfumes, cosmetics, and sacred unguents (Jones et al. 2006). Sandalwood oil has various biological activities, such as antiviral, antibacterial (Benencia and Courreges 1999) and antitumor activities (Kim et al. 2006). Sandalwood is also used in treatment of ailments like vomiting, poisoning, eye infections and other diseases (Sindhu et al. 2010). Therefore, *S. album* has huge medicinal value. However, there have been no chloroplast genomic studies on *S. album*.

Herein, we reported and characterized the complete *S. album* plastid genome (MN106256). One *S. album* individual (specimen number: 201807061) was collected from Puwen, Yunnan Province of China (22°25′39″N, 101°6′46″E). The specimen is stored at Yunnan Academy of Forestry Herbarium, Kunming, China and the accession number is YAFH0012863. DNA was extracted from its fresh leaves using DNA Plantzol Reagent (Invitrogen, Carlsbad, CA).

Paired-end reads were sequenced by using Illumina HiSeq system (Illumina, San Diego, CA). In total, about 31.1 million high-quality clean reads were generated with adaptors trimmed. Aligning, assembly, and annotation were conducted by CLC de novo assembler (CLC Bio, Aarhus, Denmark), BLAST, GeSeq (Tillich et al. 2017), and GENEIOUS v 11.0.5 (Biomatters Ltd, Auckland, New Zealand). To confirm the phylogenetic position of *S. album*, other eight species of order *Santalales* from NCBI were aligned using MAFFT v.7 (Katoh and Standley 2013). The Auto algorithm in the MAFFT alignment software was used to align the eleven complete genome sequences and the G-INS-i algorithm was used to align the partial complex sequences. The maximum-likelihood (ML) bootstrap analysis was conducted using RAxML (Stamatakis 2006); bootstrap probability values were calculated from 1000 replicates. *Boea hygrometrica* (JN107811) and *Rehmannia glutinosa* (MG977439) were served as the out-group.

The complete *S. album* plastid genome is a circular DNA molecule with the length of 144,101 bp, contains a large single copy region (LSC) of 83,796 bp and a small single copy region (SSC) of 11,277 bp, which were separated by a pair of inverted repeats (IR) regions of 24,514 bp. The overall GC content of the whole genome is 38.0%, and the corresponding values of the LSC, SSC, and IR regions are 35.9%, 31.4%, and 43.1%, respectively. The plastid genome contained 123 genes, including 80 protein-coding genes, 8 ribosomal RNA genes, and 35 transfer RNA genes. Phylogenetic analysis showed that *S. album* and *Osyris alba* clustered in a unique clade in *Santalales* order (Figure 1). The determination of the complete plastid genome sequences provided new molecular data to illuminate the order *Santalales* evolution.

**Figure 1.**
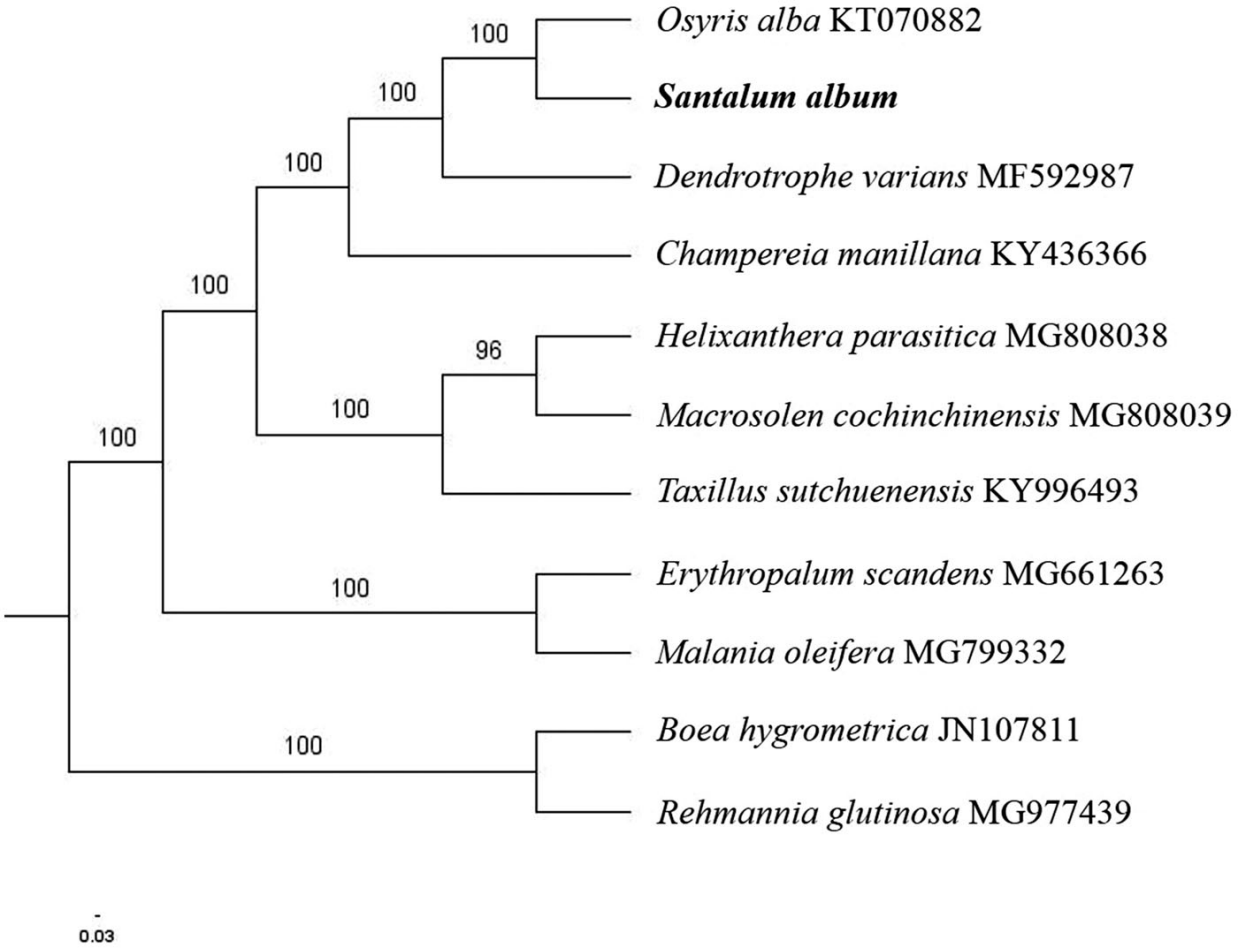
The maximum-likelihood tree based on the nine chloroplast genomes of order *Santalales*. The bootstrap value based on 1000 replicates is shown on each node.
